# Favorable Postoperative Outcomes After Transvaginal Mesh Surgery Using a Wide-Arm ORIHIME® Mesh

**DOI:** 10.7759/cureus.53388

**Published:** 2024-02-01

**Authors:** Kenji Kuroda, Koetsu Hamamoto, Kazuki Kawamura, Ayako Masunaga, Hiroaki Kobayashi, Akio Horiguchi, Keiichi Ito

**Affiliations:** 1 Department of Urology, National Defense Medical College, Tokorozawa, JPN

**Keywords:** prolapse recurrence, female urology, mesh arm width, transvaginal mesh surgery, pelvic organ prolapse

## Abstract

Introduction

Transvaginal mesh surgery (TVM) is an effective treatment option for pelvic organ prolapse (POP). Although ORIHIME®, the only available mesh product, is thin, soft, and easy to handle, it has the disadvantages of sliding off or mildly adhering to the surrounding tissues. The current study compared the efficacy of using wide-arm ORIHIME (Kono Seisakusho, Japan, Tokyo), non-wide arm ORIHIME, Gynemesh PS (Johnson and Johnson, Japan, Tokyo), and Polyform (Boston Scientific Japan, Japan, Tokyo) meshes for TVM.

Methods

The study included 116 patients who underwent TVM (Prolift with Gynemesh PS (n = 14); Elevate with Polyform (n = 43); Uphold with non-wide-arm ORIHIME^ ^(n = 24); Uphold with wide-arm ORIHIME^ ^(n = 35)) at our hospital. Pre- and post-surgical changes in symptoms were measured using questionnaires and 60-minute pad weight testing and compared by mesh type and surgical methods used.

Results

The residual urine volume, 60-minute pad weight testing, international prostate symptom score (IPSS), overactive bladder symptom score (OABSS), and international consultation on incontinence questionnaire-short form score (ICIQ-SF) significantly improved one year postoperatively in the TVM with the wide-arm ORIHIME group. Comparison of pre and one-year postoperative findings by mesh type and surgical methods used showed no significant differences in the 60-minute pad test, IPSS, Quality of Life (QOL), OABSS, and urinary incontinence in daily life scores, and improvement in residual urine volume, ICIQ-SF, and mesh exposure and POP recurrence rates in the TVM with the wide-arm ORIHIME group.

Conclusion

TVM with wide-arm ORIHIME had better postoperative outcomes compared to TVM with other mesh products.

## Introduction

Pelvic organ prolapse (POP) is defined as the descent of one or more parts of the anterior vaginal wall, posterior vaginal wall, uterus (or cervix), or vaginal apex (vaginal vault or cuff scar after hysterectomy). Approximately 50% of all women aged >50 years are at risk of developing POP, with a lifetime prevalence of 30%-50% [1]. In addition, approximately 12% of women with a normal life expectancy undergo one or more surgeries for POP or urinary incontinence and may require reoperation at a rate of approximately 29% by the age of 80 years [2].

Robot-assisted sacrocolpopexy (RSC) has recently been recognized as the leading method of sacrocolpopexy, but in the past several years, patients with POP in Japan have been treated with laparoscopic sacrocolpopexy (LSC) or transvaginal mesh surgery (TVM) [3]. Although both LSC and TVM exhibit favorable outcomes [3,4], the former exhibits superior postoperative findings in patients with severe POP (i.e., pelvic organ prolapse quantification (POP-Q) > stage 3), suggesting that the latter is unsuitable for patients with POP-Q stage 4 [4,5]. However, the postoperative outcomes of TVM have been shown to be superior or equal to that of LSC in patients with POP-Q less than or equal to stage 3, with one study suggesting that postoperative recurrence rates were lower in patients treated with TVM as compared to those treated with LSC [6].

In Japan, TVM procedures have evolved considerably over time. Prolift-type TVM procedures were first introduced by Takeyama et al. [7]. This was followed by the introduction of Elevate-type TVM surgeries by Long et al. [8], and then Uphold-type TVM surgeries, which soon became the treatment of choice and was also used in this study [9,10]. In addition, ORIHIME® is the only mesh product available for TVM surgery.

We believe that TVM remains one of the most effective surgical treatments to cure POP. The current study examined differences in the postoperative incidence of lower urinary tract symptoms (LUTS), prolapse recurrence, and complications in patients who underwent Prolift-type TVM using Gynemesh PS, Elevate-type TVM using Polyform, Uphold-type TVM using non-wide-arm ORIHIME, or Uphold-type TVM using wide-arm ORIHIME.

## Materials and methods

Patients

One hundred and sixteen patients with POP treated with TVM at our hospital between June 2012 and March 2023 were enrolled in this study. The surgical indication was POP of stage further than or equal to 2 with symptoms such as a feeling of vaginal prolapse. Prolift-type TVM using Gynemesh PS was performed for 14 patients (Gynemesh PS group), and Elevate-type TVM using Polyform was performed for 43 patients (Polyform group). Uphold-type TVM using non-wide-arm ORIHIME or wide-arm ORIHIME was performed for 24 and 35 patients, respectively (non-wide-arm ORIHIME and wide-arm ORIHIME group).

All procedures performed in this study were in accordance with the ethical standards of the National Defense Medical College (Saitama, Japan; ID, 4219). This study protocol was accepted on August 21, 2020, by the National Defense Medical College Ethics Committee. Written informed consents were collected from all patients. Patients who underwent TVM during the period written above were considered for inclusion in this study, but patients who did not give consent to participate in this study were excluded. This study is a retrospective study based on an investigation of past medical records and is not a prospective study.

The median postoperative observation period was 12.6 (range: 6.0 - 86.3 months). The surgical durations (mean ± standard deviation) were 3.0 ± 0.8, 1.0 ± 0.3, and 1.0 ± 0.2 hours in the Gynemesh PS, non-wide-arm ORIHIME, and wide-arm ORIHIME groups, respectively, while the median surgical duration (median (minimum-maximum)) in the Polyform group was 1.5 (1.0 - 3.3) hours.

Surgical methods

The Uphold-type, Elevate-type, and Prolift-type TVM surgeries were primarily carried out in accordance with the techniques reported by Takazawa et al. [10], Long et al., and Takeyama et al., respectively [7,8]. Notably, vaginal hysterectomy was not performed in any cases. Since this is a retrospective study, no randomization was performed. Prolift-type TVM was performed for the early cases, and Uphold-type TVM with fewer puncture points and a modified technique was performed in the more recent cases.

**Figure 1:**
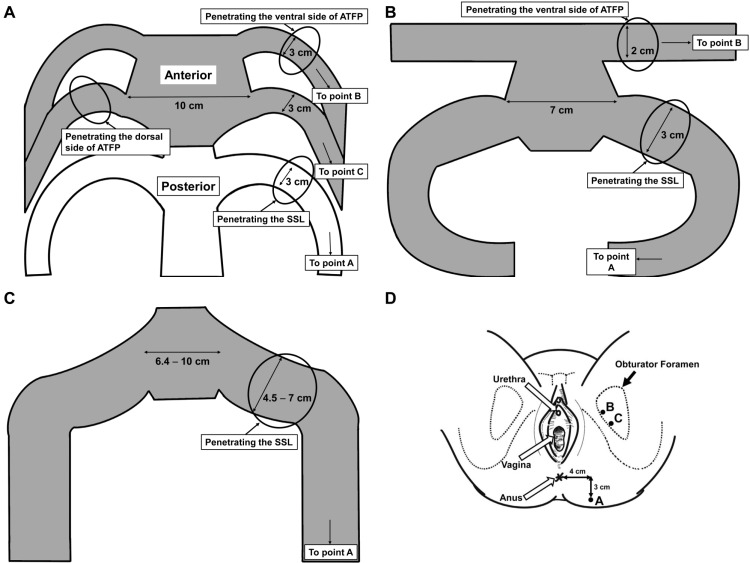
The meshes and stencil papers used for the exteriorized arms Stencil papers for Prolift-type TVM (A), Elevate-type TVM (B), and Uphold-type TVM (C). Each arm was withdrawn through the respective skin incision as described (D). These figures were created by the first author, Kuroda using Procreate (Savage Interactive Pty, Australia, Hobart). Image credits: Kenji Kuroda

During TVM, hydrodissection was first performed. Thereafter, a vertical incision was made on the anterior or posterior vaginal wall, and a lateral full-thickness blunt dissection of the pubocervical or rectovaginal fascia was carried out to reach the sacrospinous ligaments or arcus tendineus fascia pelvis (ATFP). A skin incision was then made 4 cm laterally and 3 cm inferior to the anal center (A) or the inner margin of the obturator foramen at the level of the urethra (B) and 1 cm laterally, 2 cm inferior to the point B (C) (A, B, and C: Prolift-type TVM; A and B: Elevate-type TVM; A: Uphold-type TVM). Shimada or Emmet needles threaded with nylon monofilament sutures were then used to further penetrate the sacrospinous ligaments or ATFP from the incision, targeting a point one or two finger breadths medial to the ischial spine. Thereafter, the mesh arms (Gynemesh PS (Johnson and Johnson, Japan, Tokyo), Polyform (Boston Scientific Japan, Japan, Tokyo), or ORIHIME (Kono Seisakusho, Japan, Tokyo)) were withdrawn using the nylon monofilament loops and an appropriate shape was designed, spread, and fixed under the bladder and/or over the rectum. The width of the mesh arm was approximately 3 cm in the Gynemesh PS and Polyform groups and approximately 4.5 cm to 7 cm in the ORIHIME group. The meshes were then cut to fit each stencil paper with 2 to 6 arms (Figure 1). Finally, traction was used over the exteriorized arms to ensure correct positioning, and the vaginal wound was closed using 2-0 Vicryl (Johnson and Johnson, Japan, Tokyo) sutures.

Assessment methods of preoperative and postoperative parameters

To assess the change in QOL at one year postoperatively, residual urine volume, 60-min pad test, international prostate symptom score (IPSS), overactive bladder symptom score (OABSS), and international consultation on incontinence questionnaire-short form (ICIQ-SF) were used. ICIQ-SF is available by logging in to the ICIQ Group website and agreeing to the terms and conditions (https://iciq.net/register).

Prolapse recurrence was defined as the most dependent portion being at the POP-Q stage further than or equal to 2, which means that the most distal prolapse portion is greater than or equal to 1 cm from the hymen plane, according to Takazawa et al. [10]. Urinary incontinence occurrence was defined as a daily life disturbance with incontinence. Mesh exposure was vaginally and/or visually examined using a vaginal scope.

Statistical analysis

The Wilcoxon’s signed-rank test was used to compare preoperative and 3, 6, and 12-month postoperative values within each group while the Pearson’s chi-square test was used to compare preoperative and one-year postoperative outcomes by mesh type and TVM method used. Statistical analysis was performed using JMP PRO version 17 (SAS Institute, Cary, NC). A p-value < 0.05 was considered statistically significant.

## Results

Table 1 shows clinical data such as age, BMI, POP-Q stage, details of POP, previous laparotomies, blood loss, and operative time, and major complications. Intraoperative bladder injury was observed in two patients and peritoneal injury in two patients; however, no serious postoperative complications were observed. All of them were rated as Clavien-Dindo classification grade 1 [11].

**Table 1:** Clinical characteristics of the patients AW: arm width. BMI: body mass index. POP-Q: pelvic organ prolapse quantification. VVP: vaginal vault prolapse. Numbers ± numbers mean ± standard deviation (SD), and the number (number - number) means the median (minimum-maximum). If the population was normally distributed, the values were expressed as mean ± SD; if the population was non-normally distributed, the values were expressed as the median (minimum-maximum).

	Gynemesh PS group (n = 14)		Polyform group (n = 43)		ORIHIME AW <6 cm group (n = 24)		ORIHIME AW >6 cm group (n = 35)	
Age (y)	69.9 ± 5.9		74.9 ± 6.3		78.5 (55 - 86)		75.0 ± 7.4	
BMI (kg/m^2^)	24.8 ± 3.4		24.3 (19.3 - 39.6)		23.7 ± 3.6		25.3 ± 3.9	
POP-Q stage	Stage 3	n	Stage 3	n	Stage 3	n	Stage 3	n
	Cystocele	6	Cystocele	21	Cystocele	11	Cystocele	23
	Cystocele + Hysterocele	4	Cystocele + Hysterocele	16	Cystocele + Hysterocele	5	Cystocele + Hysterocele	4
	Cystocele + VVP	2	Cystocele + VVP	5	Cystocele + VVP	2	Cystocele + VVP	5
	Hysterocele	1					Hysterocele	1
	Cystocele + Rectocele	1						
	Stage 4	n	Stage 4	n	Stage 4	n	Stage 4	n
		0	Cystocele + VVP	1	Cystocele + VVP	3	Cystocele + VVP	1
					Cystocele + Hysterocele	3	Cystocele + Hysterocele	1
Previous laparotomy	0 (0 - 2)		0 (0 - 2)		1 (0 - 3)		0 (0 - 4)	
Blood loss (mL)	27.5 (1 - 434)		63 (0 - 430)		25 (4 - 250)		20 (3 - 128)	
Operative time (h)	3.0 ± 0.8		1.5 (1.0 - 3.3)		1.0 ± 0.3		1.0 ± 0.2	
Major complications	Bladder injury (n = 2)		0		0		Peritoneal injury (n = 1)	
	Peritoneal injury (n = 1)							

**Figure 2:**
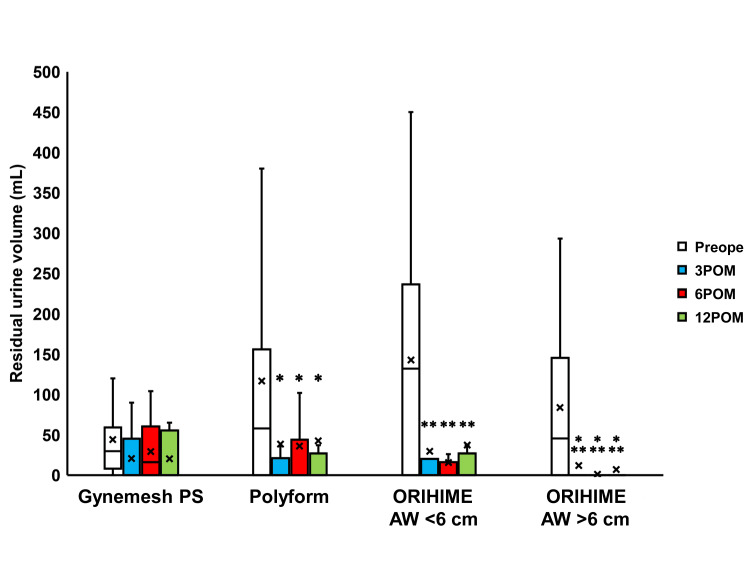
Change in the residual urine volume before and after surgery Box plots reveal a significant decrease in residual urine volume 3 to 12 months after surgery (3 to 12 POM) in the Polyform group, ORIHIME arm width (AW) <6 cm group, and ORIHIME AW >6 cm group. *, **, *** = significantly different from the preoperative period (Preope) (all p < 0.01, all p < 0.001, all p < 0.0001, respectively).

**Figure 3:**
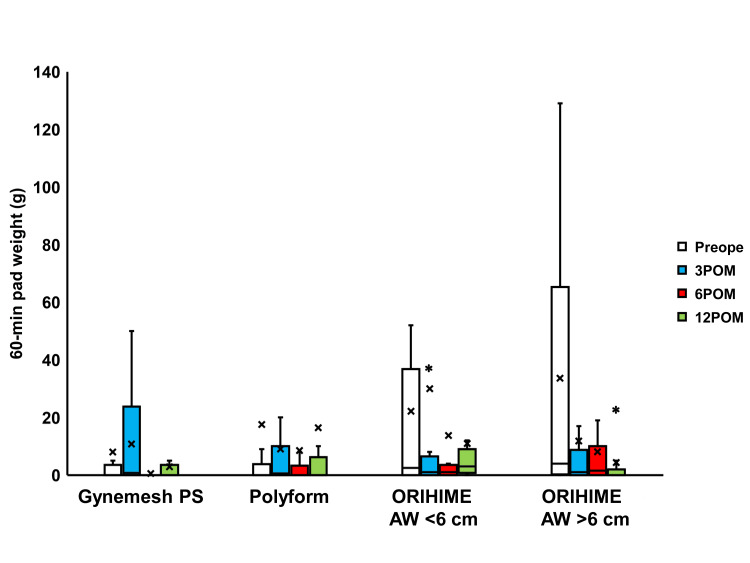
Change in the 60-min pad weight test before and after surgery Box plots reveal a significant change in the 60-min pad weight test 3 months after surgery (3 POM) in the ORIHIME arm width (AW) <6 cm group, and 12 months after surgery (12 POM) in the ORIHIME AW >6 cm group. * = significantly different from the preoperative period (Preope) (p = 0.0391, p = 0.0208, respectively).

Compared to preoperative values, the residual urine volume was seen to be significantly decreased 3, 6, and 12 months after surgery in the Polyform, ORIHIME arm width (AW) <6 cm, and ORIHIME AW >6 cm groups (p < 0.01; Figure 2). However, the 60-minute pad weight test findings did not significantly change postoperatively in the Gynemesh PS and Polyform groups while a significant decrease was observed three months postoperatively in the ORIHIME AW <6 cm group (p = 0.0391) and 12 months postoperatively in the ORIHIME AW >6 cm group (p = 0.0208; Figure 3).

**Figure 4:**
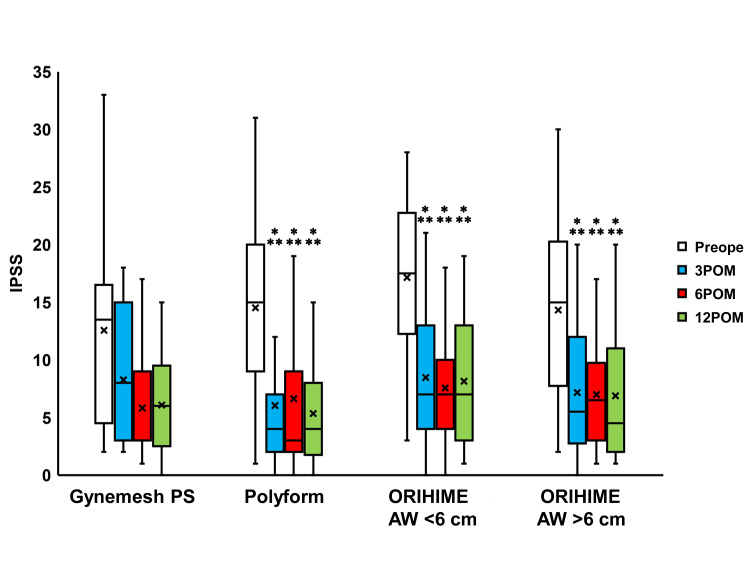
Change in IPSS before and after surgery Box plots reveal a significant change 3-12 months after surgery (3 to 12 POM) compared to preoperative scores in IPSS in the Polyform group, ORIHIME arm width (AW) <6 cm group, and ORIHIME AW >6 cm group. *** = significantly different from the preoperative period (Preope) (all p < 0.0001). IPSS: international prostate symptom score

**Figure 5:**
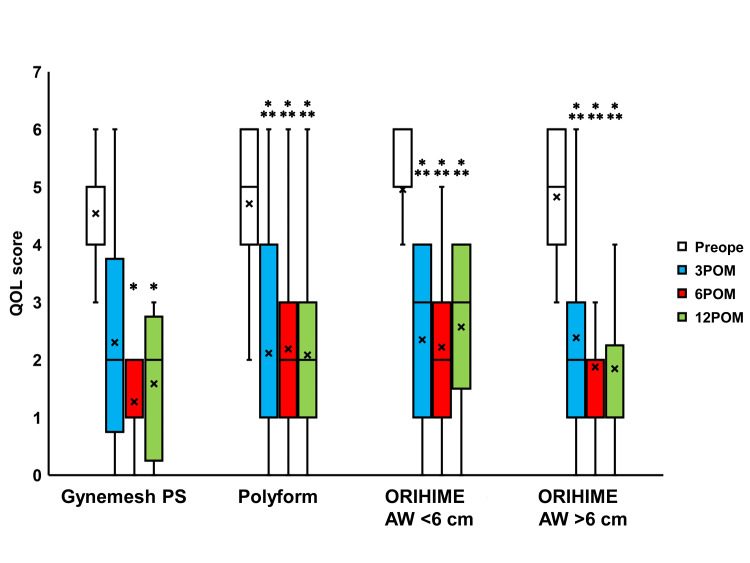
Change in QOL score before and after surgery Box plots reveal a significant change in QOL score at 6 and 12 months after surgery (6, 12 POM) in the Gynemesh PS group, and 3 to 12 months after surgery (3 to 12 POM) in the Polyform group, ORIHIME arm width (AW) <6 cm group, and ORIHIME AW >6 cm group compared to preoperative scores (all p < 0.0001). *, *** = significantly different from preoperative period (Preope) (both p = 0.0078, all p < 0.0001, respectively). QOL: quality of life

**Figure 6:**
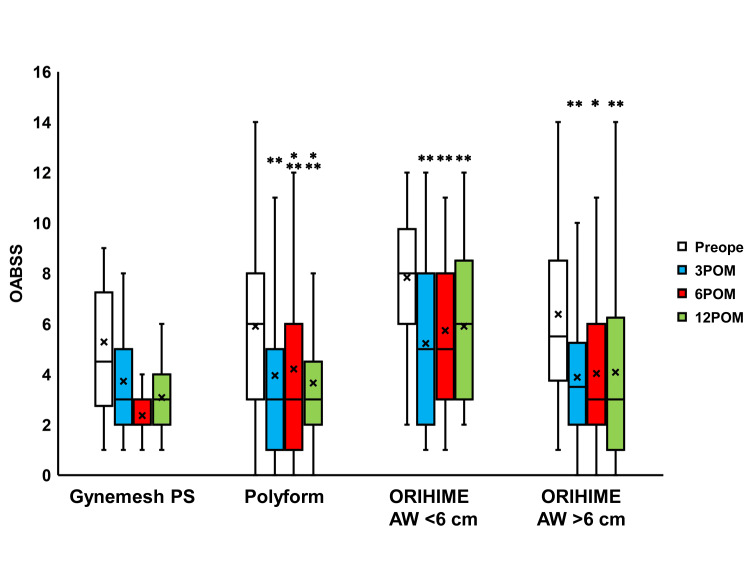
Change in OABSS before and after surgery Box plots reveal significant improvement for OABSS 3 to 12 months after surgery (3 to 12 POM) in the Polyform group, ORIHIME arm width (AW) <6 cm group, and ORIHIME AW >6 cm group. *, **, *** = significantly different from preoperative period (Preope) (p < 0.05, all p < 0.01, all p < 0.0001, respectively). OABSS: overactive bladder symptom score

**Figure 7:**
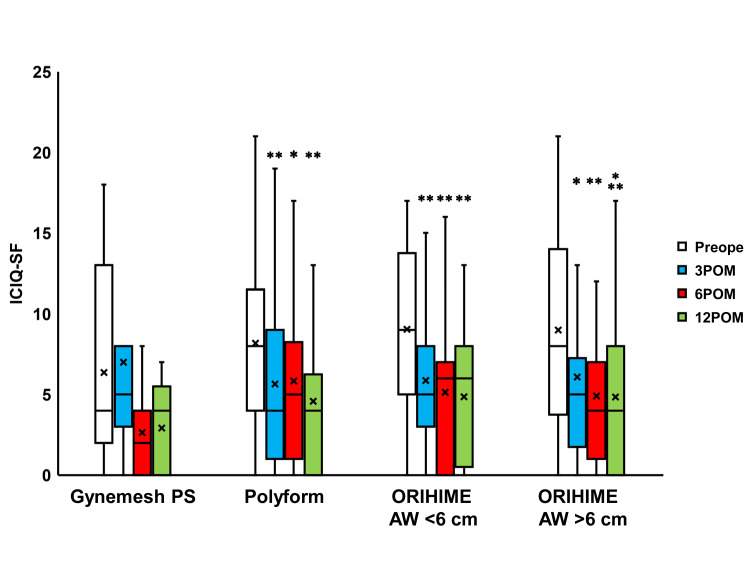
Change in ICIQ-SF before and after surgery Box plots reveal a significant improvement in ICIQ-SF found 3 to 12 months after surgery (3 to 12 POM) in the Polyform group, ORIHIME arm width (AW) <6 cm group, and ORIHIME AW >6 cm group. *, **, *** = significantly different from either of the preoperative periods (Preope) (all p < 0.05, all p < 0.01, p < 0.0001, respectively). ICIQ-SF: incontinence questionnaire-short form

Compared to the preoperative values, IPSS scores were seen to significantly change 3, 6, and 12 months after surgery in the Polyform, ORIHIME AW <6 cm, and ORIHIME AW >6 cm groups (p < 0.0001; Figure 4) while the Quality of Life (QOL) scores significantly changed 6 and 12 months after surgery in the Gynemesh PS group (both p = 0.0078) and 3 to 12 months after surgery in the Polyform, ORIHIME AW <6 cm group, and ORIHIME AW >6 cm group (p < 0.0001; Figure 5). The OABSS and ICIQ-SF scores significantly improved 3 to 12 months after surgery in the Polyform, ORIHIME AW <6 cm, and ORIHIME AW >6 cm groups (all p < 0.05; Figure 6 and Figure 7, respectively).

When comparing the results at one year postoperatively to preoperative conditions according to the mesh used, no significant differences were found in the 60-min pad test, IPSS total score, QOL score, and OABSS. However, the residual urine volume was significantly improved in the ORIHIME AW >6 cm group compared to the ORIHIME AW <6 cm group (p = 0.0335) while the ICIQ-SF score was significantly better in the ORIHIME AW >6 cm group compared to the Gynemesh PS and Polyform groups (p = 0.0018 and 0.0197, respectively; Table 2).

**Table 2:** Association between mesh used in TVM and postoperative results at one year compared to preoperative conditions AW: arm width. TVM: transvaginal mesh. IPSS: international prostate symptom score. QOL: quality of life score. OABSS: overactive bladder symptom score. ICIQ-SF: international consultation on incontinence questionnaire-short form. * Pearson’s chi-square test was used.

	1. Gynemesh PS group	2. Polyform group	3. ORIHIME AW <6 cm group	4. ORIHIME AW >6 cm group		p value
Residual urine volume					1 vs 4	0.1007
Worse	3 (21.4%)	7 (16.3%)	6 (25.0%)	2 (5.7%)	2 vs 4	0.1463
Better or no change	11 (78.6%)	36 (83.7%)	18 (75.0%)	33 (94.3%)	3 vs 4	0.0335*
60-min pad test					1 vs 4	0.5582
Worse	3 (21.4%)	10 (23.3%)	8 (34.8%)	4(14.3%)	2 vs 4	0.3532
Better or no change	11 (78.6%)	33 (76.7%)	15 (65.2%)	24 (85.7%)	3 vs 4	0.086
IPSS					1 vs 4	0.14
Worse	2 (14.3%)	6 (14.0%)	2 (8.3%)	1 (2.9%)	2 vs 4	0.0951
Better or no change	12 (85.7%)	37 (86.0%)	22 (91.7%)	33 (97.1%)	3 vs 4	0.3611
QOL					1 vs 4	0.1153
Worse	1 (7.1%)	3 (7.0%)	1 (4.2%)	0 (0%)	2 vs 4	0.1162
Better or no change	13 (92.9%)	40 (93.0%)	23 (95.8%)	34 (100%)	3 vs 4	0.2299
OABSS					1 vs 4	0.4735
Worse	2 (14.3%)	7 (18.6%)	3 (12.5%)	8 (23.5%)	2 vs 4	0.5969
Better or no change	12 (85.7%)	36 (81.4%)	21 (87.5%)	26 (76.5%)	3 vs 4	0.2913
ICIQ-SF					1 vs 4	0.0018*
Worse	5 (35.7%)	9 (20.9%)	4 (16.7%)	1 (2.9%)	2 vs 4	0.0197*
Better or no change	9 (64.3%)	34 (79.1%)	20 (83.3%)	33 (97.1%)	3 vs 4	0.0666

No significant differences in the incidence of urinary incontinence were observed between patients in the Gynemesh PS, Polyform, ORIHIME AW <6 cm, and ORIHIME AW >6 cm groups. However, the Gynemesh PS and ORIHIME AW >6 cm groups differed significantly in terms of mesh exposure while the ORIHIME AW <6 cm and ORIHIME AW >6 cm groups differed significantly in terms of prolapse recurrence rates (p = 0.0202 and 0.0098, respectively; Table 3).

**Table 3:** Association between mesh used in TVM and the presence of incontinence, mesh exposure, prolapse recurrence AW: arm width. TVM: transvaginal mesh. * Pearson’s chi-square test was used.

		1. Gynemesh PS group	2. Polyform group	3. ORIHIME AW <6 cm group	4. ORIHIME AW >6 cm group		p value
Incontinence	Present	4 (28.6%)	8 (18.6%)	3 (12.5%)	4 (11.4%)	1 vs 4	0.1425
	Absent	10 (71.4%)	35 (81.4%)	21 (87.5%)	31 (88.6%)	2 vs 4	0.3823
						3 vs 4	0.9005
Mesh exposure	Present	5 (35.7%)	1 (2.3%)	0 (0%)	3 (8.6%)	1 vs 4	0.0202*
	Absent	9 (64.3%)	42 (97.7%)	24 (100%)	32 (91.4%)	2 vs 4	0.2136
						3 vs 4	0.1410
Prolapse recurrence	Present	0 (0%)	4 (9.3%)	6 (25.0%)	1 (2.9%)	1 vs 4	0.5228
	Absent	14 (100%)	39 (90.7%)	18 (75.0%)	34 (97.1%)	2 vs 4	0.2477
						3 vs 4	0.0098*

## Discussion

TVM with ORIHIME resulted in significantly improved LUTS 3 months postoperatively when compared to TVM with Polyform (Figure 2, 4-7). 60-min pad weight decreased significantly after 3 months of surgery in the ORIHIME AW <6 cm group and 12 months of the ORIHIME AW >6 cm group (Figure 3). Furthermore, the ORIHIME AW >6 cm group exhibited greater improvement in residual urine volume one year postoperatively when compared to the ORIHIME AW <6 cm group while the ICIQ-SF scores were significantly improved in the ORIHIME AW >6 cm group compared to the Gynemesh PS and Polyform groups (Table 2). Mesh exposure and prolapse recurrence rates were significantly lower in the ORIHIME AW >6 cm group compared to the Gynemesh PS and ORIHIME AW <6 cm groups, respectively (Table 3).

Regarding TVM, in 2019, the US Food and Drug Administration (FDA) ordered all manufacturers of surgical meshes intended for transvaginal repair of anterior vaginal wall prolapse to cease selling and distributing their products, effective immediately. Subsequently, this announcement led to much controversy and raised ongoing questions about whether TVM surgery is appropriate for POP. However, in Japan, TVM surgery has been reported to have successful outcomes and low mesh-related complications in addition to a low rate of prolapse recurrence after surgery [12,13]. In addition, a significant improvement in QOL was observed in patients treated with TVM. Notably, their studies indicated that their minimal mesh approach to vaginal treatments should be considered as an option for managing POP [10,14,15].

Gynemesh PS was the first synthetic polypropylene (PP) mesh product used for POP surgery in Japan, although its use in TVM was prohibited in April 2014. Then another PP soft mesh, Polyform was available at that time but was banned for TVM use in May 2019 because of the increasing frequency of mesh-related postoperative complications in some countries except Japan. Thereafter, polytetrafluoroethylene (PTFE) mesh, ORIHIME was approved and used in this series of surgeries. In fact, some studies evaluated the clinical outcome and QOL of the postoperative course using PP mesh and ORIHIME mesh in the surgery for POP. They concluded that there was no significant difference between both mesh materials in the short term, and PTFE mesh might be one of the most suitable mesh materials for pelvic floor reconstruction [16,17].

Currently, ORIHIME is the only mesh product available for TVM surgery, emphasizing the need for developing ways to prevent POP recurrence. Kuwata et al. found that ORIHIME mesh exhibited milder adhesion with the surrounding tissues, and the high recurrence rates could primarily be attributed to the mesh slipping off the fixation site as opposed to the insufficient anchoring observed with PP meshes [16]. Therefore, the mesh AW was increased to at least 4.5 cm at first to prevent it from slipping off as this width was considered to be sufficiently wider (1.5×) than the 3 cm width of Polyform. Eventually, the AW was further increased to 7 cm, and no recurrence has been observed so far. However, future studies should examine mesh AW further to confirm this.

Since Gynemesh PS, as well as Polyform, were allowed for TVM surgery, minimal mesh surgery has been preferred to avoid mesh-related complications, and favorable postoperative outcomes were obtained [10,15,18]. However, minimal mesh surgery with PTFE is not practical, as the findings of this study showed increased POP recurrence when using PTFE meshes with the same AW as PP. Using techniques with low POP recurrence rates can help maintain stable bladder support and improve LUTS, with several studies showing that an improvement in LUTS was significantly associated with lower recurrence rates [3,10,18]. The current study also found a significant decrease in residual urine volume and ICIQ-SF scores compared to other TVM methods, although further follow-up is necessary to confirm this.

Regarding the degree of improvement of pelvic organ prolapse, the prolapse quality of life questionnaire (P-QOL) is routinely used [19], but there were several missing values, so it was excluded from the results of the present study. Alternatively, the degree of improvement of POP was assessed by medical interview and physical examination in the lithotomy position. IPSS can be used besides male patients with benign prostatic hyperplasia and was used for the evaluation of voiding dysfunction in female patients with POP [20,21].

This study has some limitations. First, the number of patients is relatively small. Although the number of patients treated with Prolift-type TVM was particularly small, they were retained in the study sample to enable the identification of effective TVM techniques. Second, long-term results over several years are not currently available. Third, this is a single-center, retrospective study. However, within the limitations of this study, it can be concluded that wide-arm ORIHIME represents a suitable treatment option.

## Conclusions

TVM using wide-arm OIHIME was associated with an improvement in postoperative LUTS and lower complication and recurrence rates compared to TVM using Gynemesh PS, Polyform, or non-wide arm ORIHIME. This suggests that when ORIHIME is the only available option for TVM surgery, making wide-arm adjustments may be a better treatment strategy.
